# Investigating interbrain synchrony under teamwork disruption: an fNIRS hyperscanning study

**DOI:** 10.1186/s12993-026-00320-6

**Published:** 2026-02-08

**Authors:** Coralie Réveillé, Grégoire Vergotte, Gérard Dray, Pierre-Antoine Jean, Pierre Jean, Simon Pla, Stephane Perrey, Grégoire Bosselut

**Affiliations:** EuroMov Digital Health in Motion, Univ. Montpellier, IMT Mines Alès, Montpellier and Alès, France

**Keywords:** Interbrain synchrony, Teamwork, Disruption, Team adaptation, fNIRS hyperscanning

## Abstract

**Background:**

Teams are inherently adaptive entities that continuously adapt to changes or disruptions in their tasks or environments. During collaboration, interbrain synchrony (IBS) emerges, reflecting the temporal alignment of neural activity between team members. Building on this, IBS has been proposed as a potential marker of teamwork, suggesting that IBS should be sensitive to changes in teamwork.

**Purpose:**

The present study investigated whether IBS is sensitive to changes in teamwork. We hypothesized that disruptions in teamwork would be accompanied by alterations in IBS dynamics.

**Methods:**

Ninety-eight healthy adults (mean age = 22.5 ± 3.22 years; 69 females, 65.1%) were assigned to forty-nine dyads. Each pair completed a 20-minute computer-based navigation task while their brain activity was simultaneously recorded using fNIRS hyperscanning. Dyads in the experimental group encountered an unexpected increase in task difficulty midway through the task, whereas those in the control group completed the task without disruption. We examined three features of IBS - its overall level, temporal slope trajectory, and the temporal recurrence patterns.

**Results:**

Control analyses confirmed that IBS reliably emerged during the task (χ²(1) = 50.24, p < .001) and that the experimental manipulation successfully disrupted teamwork, as reflected in altered team behavioral responses in communication (χ²(1) = 8.48, p = 0.004) and performance (χ²(1) = 24.99, p < .001). Nevertheless, no evidence was found for disruption-related changes in IBS across the three features examined (all Time x Group interactions p > .05.

**Conclusion:**

These findings raise the possibility that IBS may reflect a stable collective state rather than a reactive one, thereby challenging its interpretation as a direct marker of teamwork. Methodological considerations, including the operationalization of IBS, are also discussed as potential explanations for the lack of observed change in IBS.

**Supplementary Information:**

The online version contains supplementary material available at 10.1186/s12993-026-00320-6.

## Highlights

- A sudden increase in task difficulty was used to disrupt teamwork.
- Three interbrain synchrony features (level, slope, %DET) were used to assess temporal dynamics.
- Interbrain synchrony remained unchanged despite teamwork disruption.
- Teamwork adaptation was observed in communication but not in interbrain synchrony.
- Findings question whether interbrain synchrony reflects reactive or stable team state.

**Supplementary Information:**

The online version contains supplementary material available at 10.1186/s12993-026-00320-6.

## Background

Teams are adaptive entities that operate in dynamic environments [3]. Their ability to continuously adjust to evolving task demands is essential for maintaining performance, particularly in the face of unexpected events or disruptions. For example, flight crews that engaged in adaptive behaviors committed fewer errors [83, 110, 114, 122]. It is therefore important to determine whether IBS can capture dynamic changes in collaborative performance.

Teamwork is a social interaction during which two or more interdependent individuals interact with one another to achieve a shared goal [85, 107]. During teamwork, collective phenomena known as *teamwork emergent states* arise [65], and manifest across three ABC levels : affective, behavioral and cognitive levels [12, 77]. For example, the affective level (“A”) refers to phenomena such as team cohesion or team mood; the behavioral level (“B”) encompasses patterns of communication and action coordination; and the cognitive level (“C”) refers to team cognition—that is, how task-relevant knowledge is mentally organized, represented, and shared among team members, as in shared mental models or transactive memory systems [12, 28]. Teams are thus conceptualized as entities that feel, act and think as a cohesive unit [47].

Rather than being static, teamwork interaction is inherently a dynamic phenomenon [3], with *dynamics* broadly referring to phenomena that change over time [75]. Within this perspective, the affective, behavioral, and cognitive emergent states that characterize teamwork are not static but rather vary over time, following three distinct types of temporal dynamics [30, 64, 75].

First, *developmental dynamics* describes how teams mature over time. It assumes that teams go through different stages, and that an earlier stage prepares the ground for the next stage [67, 120]. Second, *cyclic or episodic dynamics* underlies that teams engage in distinct activities at different times, with these activities recurring in a predictable and cyclical pattern (e.g., [77]). It suggests that teams engage in recurring cycles of activities, or “episodes” (i.e., distinct periods where performance accumulates and feedback becomes available, [81]). Finally, *event based dynamics* assumes that specific circumstance may disrupt the normal course of team’s activities and functioning [93], triggering changes in the ABCs of teamwork [30, 75].

A disruption is a specific event that, due to its nature or intensity, compels the team to adopt a new mode of operation [41]. A critical shift in the environment [56], creates a change in task demand characterized by novelty or increased difficulty [11], that constitutes a functional challenge for the team [16, 115]. The team is thus forced to adapt in order to be able to continue performing its task while striving to maintain the highest possible level of performance [16].

Team adaptation refers to the outcome of an adaptation process through which team modifies its functioning. Team adaptation is thus the enduring observable changes in team functioning in response to a disruption [6, 83]. These outcomes can be measured through the appearance of a stable new – or the modification of existing – ABCs team-level phenomena that address the demands of a changing environment [6, 16].

Research has demonstrated that a disruptive event may alter not only team performance, but also communication behaviors within the team – the “B” from the ABC team level phenomena – but the nature of the change depends on the type of disruption. For example, Barth et al. [9] observed that in a surgical team facing an unforeseen and sudden increase in task difficulty (N_team_ = 1), both the structure and content of the communication network changed. Burtscher et al. [17] found an increase in the amount of communication after an unforeseen and non-routine event in anesthesia dyads (N_teams_ = 15), suggesting that more communication helped to coordinate actions. Conversely, Entin and Serfaty [34] and Grote et al. [48] found a decrease in the amount of communication following a sudden increase in workload, as team members prioritized conserving resources for their individual tasks in naval teams (*N* = 6) and by in aeronautical crews (N_teams_ = 42), respectively.

These studies provide evidence that disruptions that increase task difficulty leads to changes in team functioning. However, their reliance on external observers limits the scope of analysis to easily identifiable collective phenomena, such as behaviors, offering little insight into changes at the affective and cognitive levels, which are also expected to change. Research on affective and cognitive team-level phenomena traditionally employs self-reported questionnaires filled by team members. However, these tools will be impractical since they require team members either to pause the task to fill out the questionnaire, or to rely on their memory to reconstruct events if the questionnaire is administered after the task [66, 70, 82]. Moreover, both external evaluations and self-questionnaires share the limitation of relying on subjective measures.

To objectively measure the change in team functioning, wearable sensors that collect the behavioral or physiological activity from team members can be used (e.g., [30, 54, 63, 66]). Aggregating individual signals by calculating their synchrony may serve as an indicator of team-level ABC phenomena [54, 60]. Spontaneous synchrony in physiological activities (i.e., activity in autonomic nervous system, [80, 91]) – may reflect affective-level emergent states (“A”), including emotional regulation or empathy (for a review, see [98]); synchrony in observable behaviors (e.g., communication patterns, motor gestures, facial expressions) may reflect behavioral-level phenomena (“B”); and synchrony in neural activity (i.e., activity in the central nervous system) may serve as an indicator of cognitive-level emergent states (“C”, Gordon [42]; [54, 60]).

Empirical evidence suggests that some of these indicators are responsive to changes in team dynamics following a disruption. At the affective level (i.e., physiological synchrony), Hałgas et al. [54] examined changes in cardiac and electrodermal synchrony following a sudden and sustained disruption involving the redistribution of tasks among team members after an unexpected computer malfunction (N_teams_ = 30). Their findings revealed no significant changes in physiological synchrony, suggesting that such disruptions may not necessarily alter the team’s affective state. At the behavioral level, Grimm [45] and Gorman [40] provided evidence that communication—particularly the structure of turn-taking (i.e., who speaks when)—is sensitive to disruptions in teamwork. Together, these findings raise the possibility that similar disruptions may also affect interbrain synchrony, reflecting changes in the team’s cognitive-level coordination.

Interbrain synchrony (IBS) is defined as the degree of similarity between neural activities of interacting individuals [55, 112]. It is typically measured using hyperscanning paradigms, which involve the simultaneous recording of brain activity from individuals engaged in a social interaction [25, 92]. Several neuroimaging techniques can be used to investigate IBS, including functional magnetic resonance imaging (fMRI), electroencephalography (EEG), and magnetoencephalography (MEG). Functional near-infrared spectroscopy (fNIRS) has been increasingly used in IBS research because it offers a particularly suitable compromise between ecological validity and neural measurement during social interaction [25, 32, 101]. Unlike fMRI, fNIRS allows participants to sit close to one another and engage in extended, naturalistic interactions. Compared to EEG or MEG, fNIRS is less sensitive to motion artifacts and facial muscle activity associated with social interaction behaviors such as speech, while providing sufficient spatial resolution to target specific cortical regions of interest for IBS analyses [25, 102]. A growing body of hyperscanning literature, including recent reviews and meta-analyses, provides strong evidence that neural activity tends to synchronize when individuals engage in social interactions [25, 95]. This phenomenon—often referred to as neural coupling—has been observed across a wide range of interaction contexts, such as during verbal communication [61], joint musical performance [19], educational exchanges [117], close interpersonal relationships [128] and even in clinical settings [1].

Among these contexts, teamwork has received particular attention. In a recent meta-analysis, Czeszumski et al. [26] demonstrated that IBS levels are significantly higher when individuals collaborate, compared to when they perform tasks individually, are at rest, or engage in competitive scenarios. These findings support the idea that IBS reflects a collective phenomenon that specifically emerges during cooperative team activities. IBS has also been associated with key features of team functioning. In particular, it is not only influenced by team characteristics, but is also associated with collective performance and various team-level emergent states (e.g., subjective feeling of cooperation, Réveillé et al. [105]). Furthermore, IBS is most consistently observed in brain regions such as the prefrontal cortex (PFC) and the temporoparietal junction (TPJ). These areas are well known for their involvement in theory of mind and mentalizing processes [38, 108], and as they are essential to social cognition [73], IBS has been proposed as a potential candidate for measuring team cognition – the “C” in the ABC of teamwork emergent states. While the exact functional role of IBS is still debated in the field, several theoretical models converge on the idea that IBS may reflect mutual prediction [51], shared intentionality [37], or the alignment of shared mental representations [27].

Evidence suggests that IBS is sensitive to task characteristics and coordination demands (Réveillé et al. [105]). For example, fNIRS hyperscanning studies have shown higher IBS during cooperative tasks requiring greater creativity, such as joint object construction or brainstorming, compared to less demanding tasks, particularly in the PFC and TPJ [74, 84]. Similarly, tasks involving more complex interpersonal motor coordination, such as joint drawing, elicit higher IBS in the PFC than simpler coordination tasks [20]. In parallel, EEG hyperscanning studies in applied settings, such as simulated aviation tasks, have reported variations in inter-subject connectivity across task phases differing in cognitive load, with higher connectivity observed during more demanding phases (e.g., take-off and landing) compared to less demanding phases [5, 119]. However, fNIRS studies typically compare different tasks or task conditions rather than changes occurring within an ongoing task, whereas existing EEG studies often lack control conditions and sufficient spatial resolution to localize the brain areas involved. More generally, neither approach has explicitly examined the effect of a sudden and unexpected disruption occurring during collaboration. Moreover, existing studies have primarily focused on changes in overall IBS level, leaving open the possibility that other temporal dynamics of IBS may also be affected.

To fill this gap, the present study aimed to examine whether a sudden increase in task difficulty occurring during an ongoing collaborative task would be reflected in the temporal dynamics of IBS. We focused on three key features of IBS, each reflecting a distinct dimension of teamwork dynamics [75]. First, we examined the average level of IBS, as previous research has shown that it varies with task characteristics and coordination demands. Second, based on developmental dynamics theories suggesting that teamwork emergent states do not appear instantaneously but instead gradually evolve over time (e.g., cohesion, team cognition, [39, 67]), we examined the developmental trajectories of interbrain synchrony. Specifically, we analysed the temporal slope of IBS time series across the task. Finally, we explored the temporal regularity of IBS fluctuations, in line with theoretical accounts proposing that teamwork unfolds through cyclic dynamics. We expected that a sustained disruption — operationalized as a sudden and unexpected increase in task difficulty — would lead to measurable changes across these three features. However, given the limited body of prior research on the temporal dynamics of IBS, and the fact that, to our knowledge, no study has yet examined the impact of a disruption on IBS, we did not formulate specific directional hypotheses.

Addressing these questions required a neuroimaging technique compatible with prolonged, face-to-face collaboration and free verbal interaction. FNIRS was therefore selected over fMRI and EEG. FNIRS enables continuous hyperscanning during ecologically valid social interactions while providing sufficient spatial resolution to investigate cortical regions implicated in teamwork (e.g., [25, 123]). This makes it particularly well suited to examine the temporal dynamics of IBS before and after a sudden task disruption.

## Methods

### Procedure

The experimental protocol was pre-registered online on the Open Science Framework (OSF; https://osf.io/na7br/) prior to data collection. The study was conducted between January and March 2025. Participants were randomly assigned to one of two groups prior to arrival: a control group, in which teams performed the task without disruption, and an experimental group, in which teams performed the same task but encountered a disruption at a predefined time.

####  Population

We recruited 106 adult participants (mean age = 22.8 ± 3.2 years; 69% female), all native French speakers, with no reported history of neurological or psychiatric disorders and no reported auditory or visual impairments. No selection was made based on handedness. 53 teams, each composed of two participants (i.e., dyads), were formed without specification on gender composition (50% female–female, 21% male–male, 28% mixed-gender dyads). To control for potential familiarity effects [100], dyads were formed by pairing individuals who did not know each other beforehand. All participants provided written informed consent prior to the study, and the protocol was approved by the local ethics committee (UM 2025-008). Details of the sample are presented in Supplementary Material 1.

#### Experimental task

**Fig. 1:**
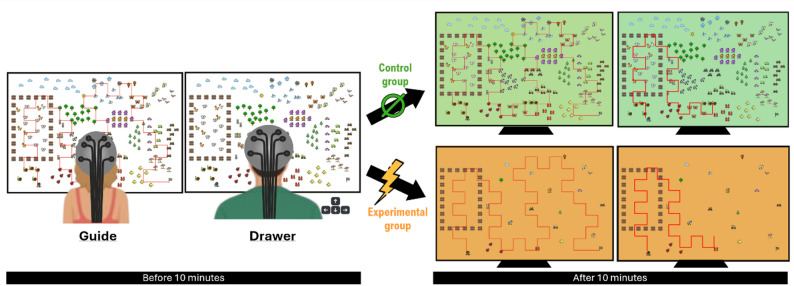
Experimental task. Left: one dyad in experimental setting: two participants, Guide is on the left side, Drawer is on the right side. Both face their screens and wear their fNIRS caps. Only the Drawer can handle keyboard’s arrows. Right top: Guide’s and Drawer’s screens after 10 min in the control group: all the 202 icons remain visible. Right bottom: Guide and Drawer’s screens after 10 min in the experimental group: only 41 icons remain visible

The experimental task consisted of a collaborative computer-based spatial navigation. Specifically, we used a customized version of the HRCR MapTasks corpus developed by the Human Communication Research Center [2], previously employed in hyperscanning studies [112]. For each dyad, participants were randomly assigned to one of two roles: Guide or Drawer. They were seated side by side, each facing their own screen, with no visual access to their partner’s display. Before the task begins, both the Guide’s and Drawer’s screens displayed identical visual elements, including a Start Point, an End Point and 202 icons. On the Guide’s screen, an additional visual element – a predefined path referred to as the “Reference Path” – connected the Start Point to the End Point and passed through several icons. This Reference Path was not be visible on the Drawer’s screen. Instead, the Drawer’s screen displayed a cursor positioned at the Start Point (Fig. 1).

Participants were instructed to work together, with the shared goal that, by the end of the task, the route displayed on the Drawer’s screen would exactly match the one already present on the Guide’s screen. At the beginning of the task, the Drawer was permitted to press one of the arrows on the keyboard in order to set the cursor in motion. The cursor moved at constant speed in a linear direction, leaving a visible trail on the Drawer’s screen. The Drawer had the capacity to modify the cursor’s trajectory at any time by using the arrow keys.

Verbal communication between participants was permitted, and precision in replicating the Reference Path was emphasized as the primary objective. The task duration was fixed at 20 min.

Prior to the main task, participants completed a 2-minute familiarization task to ensure they had a comprehensive understanding the procedure and controls. The experimental session consisted of a single trial, with 3-minutes rest periods both before and after the task. The three-minute rest periods before and after the task were used to collect additional fNIRS data, in order to minimize potential edge effects when quantifying IBS using Wavelet Transform Coherence (WTC). Indeed, since WTC analyses produce a cone of influence that distorts values at the beginning and end of the signal, it is important to ensure that these edge effects must be excluded from the task data. These edge effects affect a larger portion of the signal at the beginning and end as the frequency under consideration decreases. To ensure that all edge effects remained within the rest periods, an a priori estimation was performed. For very low frequencies (0.01 Hz), the edge effect was estimated to last 2 min and 22 s. Therefore, the rest periods were set to 3 min.

#### Experimental manipulation

For dyads in the control group, the task was carried out as described above: all icons remained visible throughout the trial, and all directional changes were made above an icon to ensure their ease of location. In contrast, for dyads in the experimental group, 10 min into the task, a disruption occurred regardless of the cursor’s position at that time: 80% of the icons disappeared from both the Guide’s and the Drawer’s screens, leaving only 41 icons (20%) visible. Importantly, the icons themselves were not relocated and the spatial layout of the environment remained unchanged. However, none of the subsequent directional changes occurred at locations marked by icons, such that the points where turns had to be performed were no longer explicitly indicated, making them more difficult to identify (Fig. 1).

To include a sufficiently impactful disruption for the experimental group, the intensity of the disruption was tested and adjusted using four pilot dyads prior to the start of the main experiment. Participants involved in these pilot trials were not eligible to take part in the main experiment.

### Interbrain synchrony measurement and analysis

The guidelines provided by Nguyen et al. [96] were used to measure and quantify IBS.

#### Brain activity measurement

Brain activity was measured with functional near infrared spectroscopy (fNIRS), using NIRScout associated with NIRStar software version 15.3 (NIRx GmbH, Berlin, Germany). Emitters and receivers were spaced approximately 30 mm from each other [103]. The sampling rate was 7.81 Hz and the wavelengths were 760 nm for deoxyhemoglobin and 850 nm for oxyhemoglobin.

**Fig. 2:**
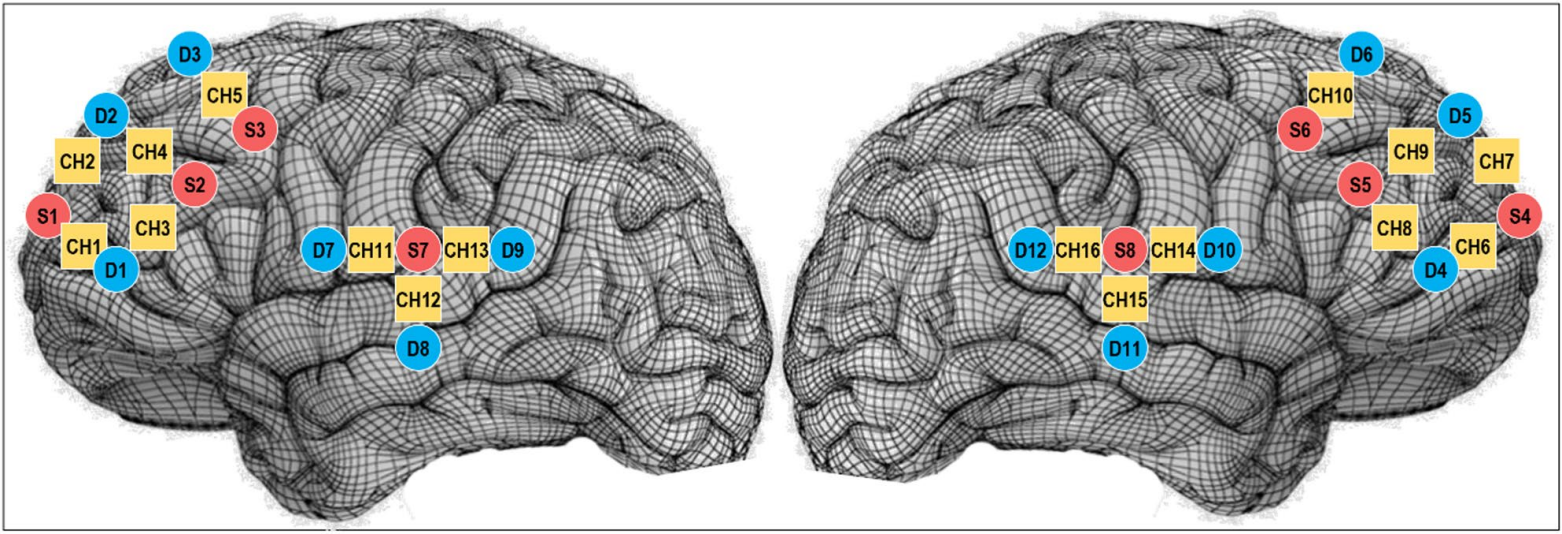
3D visualization of channels of interest covering the left and right frontopolar prefrontal and temporo-parietal junctions. Left panel: Lateral view, left hemisphere; Right panel: Lateral view, right hemisphere. Sources are in red; detectors in blue; channels in yellow.

Twenty optodes were placed on the six main brain regions of interest (ROI): the left and right frontopolar prefrontal cortex (fp PFC), dorsolateral prefrontal cortex (dl PFC), and the temporo-parietal junction (TPJ, see Fig. 2).

- The channels 1 and 6 were, respectively, located on the left and right fpPFC;
- The channels 2, 3, 4, 5, and 7, 8, 9 and 10 were, respectively, located on the left and right dlPFC;
- The channels 11, 12, 13 and 14, 15 and 16 were, respectively, located on the left and right TPJ.

These brain regions were selected based on converging findings in the current literature indicating that IBS typically emerges in these areas during cooperative tasks involving teamwork interaction [26].

####  Pre-processing and visual quality check

fNIRS data were preprocessed in accordance with established guidelines for fNIRS hyperscanning provided by Nguyen et al. [96] and Reindl et al. [103]. They were performed using the Homer2 toolbox [59] for Matlab and home-made scripts.

**Fig. 3:**
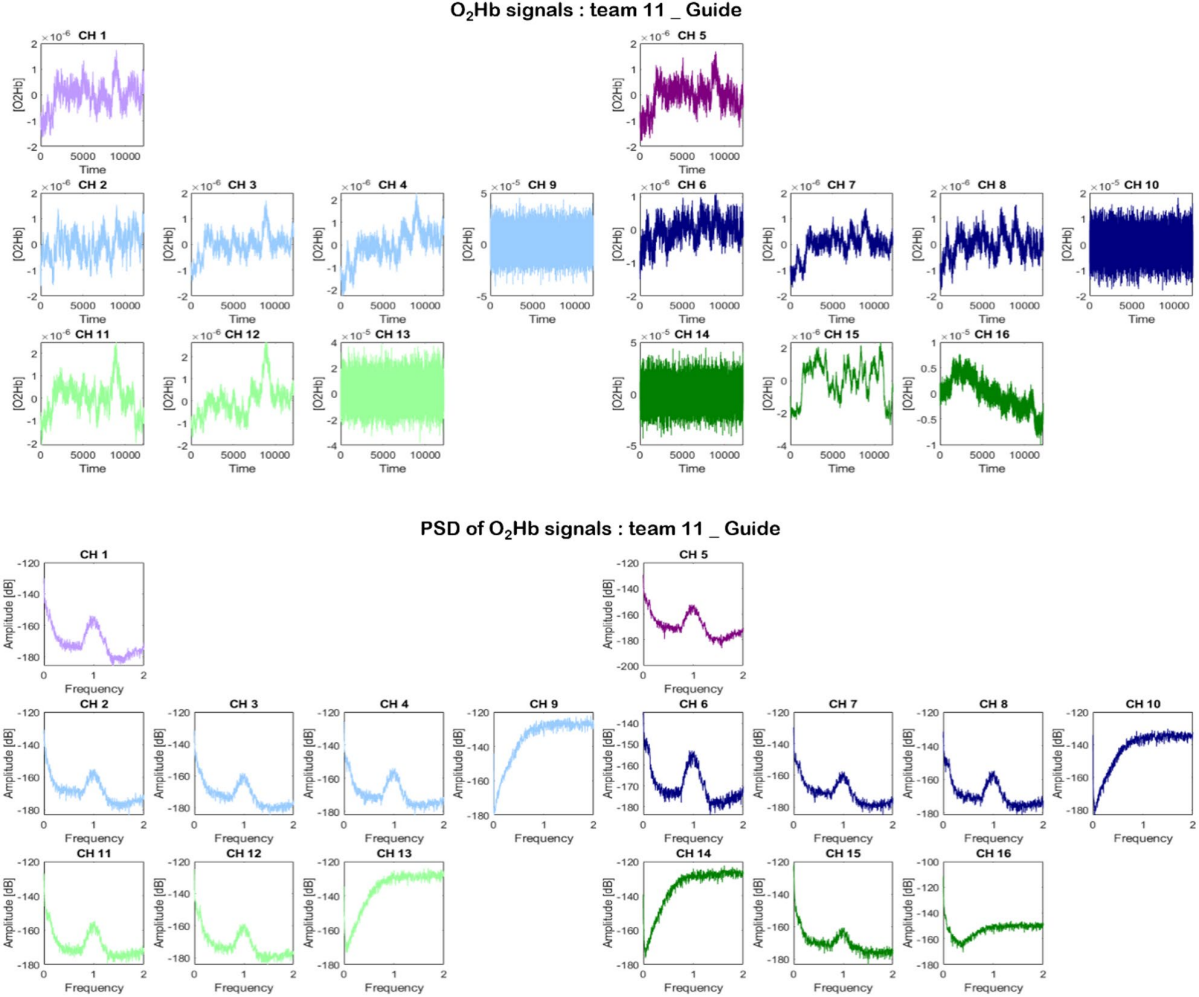
Identification of good and bad channels based on the 1 Hz peak criterion. Top panel: O₂Hb signals; Bottom panel: DSP (Density Spectral Power) of the O₂Hb signals. Graphs in the same color correspond to the same ROI. Channels 9, 10, 13, and 14 were excluded due to poor signal quality, as indicated by high-frequency noise in the O_2_Hb signals and the absence of a clear 1 Hz peak in the PSD

(1) Raw intensity data were converted to Optical Density (OD) using the function “hmrIntensity2OD”. (2) Head movement artefacts were corrected using “hmrMotionArtifactByChannel” and combining spline interpolation method [109], “hmrMotionCorrectSpline” with default parameters (SDThresh = 20, AMPThresh = 0.5, tMotion = 0.5s and tMask = 2s et *p* = .99) followed by wavelet based signal decomposition [90] “hmrMotionCorrectWavelet” with default parameter (iqr = 1.5). (3) To mitigate the influence of systemic physiological noise in the fNIRS data, a principal component analysis (PCA) was applied, preserving components accounting for 80% of the total variance [13, 58, 127]; (4) Corrected OD signals were converted to oxy (O_2_Hb) and deoxy haemoglobin (HHb) concentration data following the modified Beer-Lambert law using “hmrOD2Conc” with partial pathlength factor (ppf) set as [6 6]. (5) To ensure the quality of the data, the power spectral density of all O_2_Hb signals were then visually examined using the “pwelch” function. The presence of a peak around 1 Hz in the signal is indicative of heartbeat occurrence and is taken as an indication of good contact between the optodes and the scalp [118]. In case of the absence of the peak around 1 Hz, the channel was considered bad channel and removed from analysis (see Fig. 3). (7) fNIRS signals of oxygenated and deoxygenated hemoglobin (O₂Hb and HHb) concentrations recorded from channels assigned to the same ROI were averaged in order to obtain a hemoglobin concentration signal for each region of interest (ROI). ROIs composed exclusively of bad channels were discarded. Participants with more than one missing ROI were excluded from the dataset. Four participants were excluded, resulting in a final sample of 49 dyads: 48 dyads with all six ROIs available, and one dyad with one ROI missing. Flow chart of the data available is presented in **Supplementary Material 2.**

#### IBS quantification

IBS was quantified in accordance with the current recommendations for fNIRS hyperscanning studies [96, 103]. Three key methodological decisions guided our approach. First, we focused on the analysis of oxygenated hemoglobin (O_2_Hb) time series, given its greater sensitivity to changes in blood oxygenation and its stronger association with IBS, as supported by previous research [71, 123]. However, to provide a more comprehensive view of potential neural synchrony processes, analyses were also conducted using deoxygenated hemoglobin (HHb) signals, as recommended by Nguyen et al. [96] (see Supplementary Material 3). Second, IBS was computed between corresponding (i.e., homotopic) brain regions across participants [96, 103]. Calculating IBS between non-homotopic regions remains relatively uncommon and requires an a priori model specifying how neural activity in one brain region might influence another. Third, we used WTC to quantify IBS, as this method is widely adopted in fNIRS hyperscanning research [8, 53]. WTC enables the analysis of coherence and phase relationships between two time series across both time and frequency domains [18, 46]. The “wcoherence” function from Matlab was used ([Time, HbOGuide], [Time, HbODrawer], ‘mcc’,0). The output variables include “wcoh” (coherence values, named IBS here), frequency band “f”, and cone of influence “coi”. The WTC matrix provide IBS values as a function of frequency and time. Values corresponding to the cone of influence was excluded, since edge effect may lead to estimation bias [94, 96]. Only values corresponding to cortical frequencies (i.e., [0.01; 0.08] Hz) were conserved in order to remove frequencies of physiological noises (i.e., cardiac ~ 1 Hz, Mayer waves ~ 0.1–0.4 Hz, and respiratory ~ 0.2–0.3 Hz, [90]). While no formal guidelines currently define an optimal frequency band for IBS quantification in fNIRS hyperscanning, this range is consistent with those commonly employed in fNIRS hyperscanning studies using WTC [53].

#### IBS features quantification

The effect of the disruption was analyzed across three complementary features of IBS: level, slope, and percentage of determinism (%DET). For each dyad, the three IBS features were computed both before and after the 10-minute mark (i.e., the point at which the disruption occurred) for each ROI.

The first feature, the overall *level* of IBS, was obtained by computing the mean coherence values across both time and frequency domains.

The second and third features – slope and %DET – were derived from IBS signals, obtained by averaging coherence values across frequencies.

The second feature, the IBS *slope*, represents the trajectory of IBS over time. A positive slope indicates an increase in IBS, a negative slope indicates a decrease, and a zero slope indicates stable IBS levels. The slope was calculated by fitting a linear model to each IBS time series using the MATLAB function “polyfit”.

**Fig. 4:**
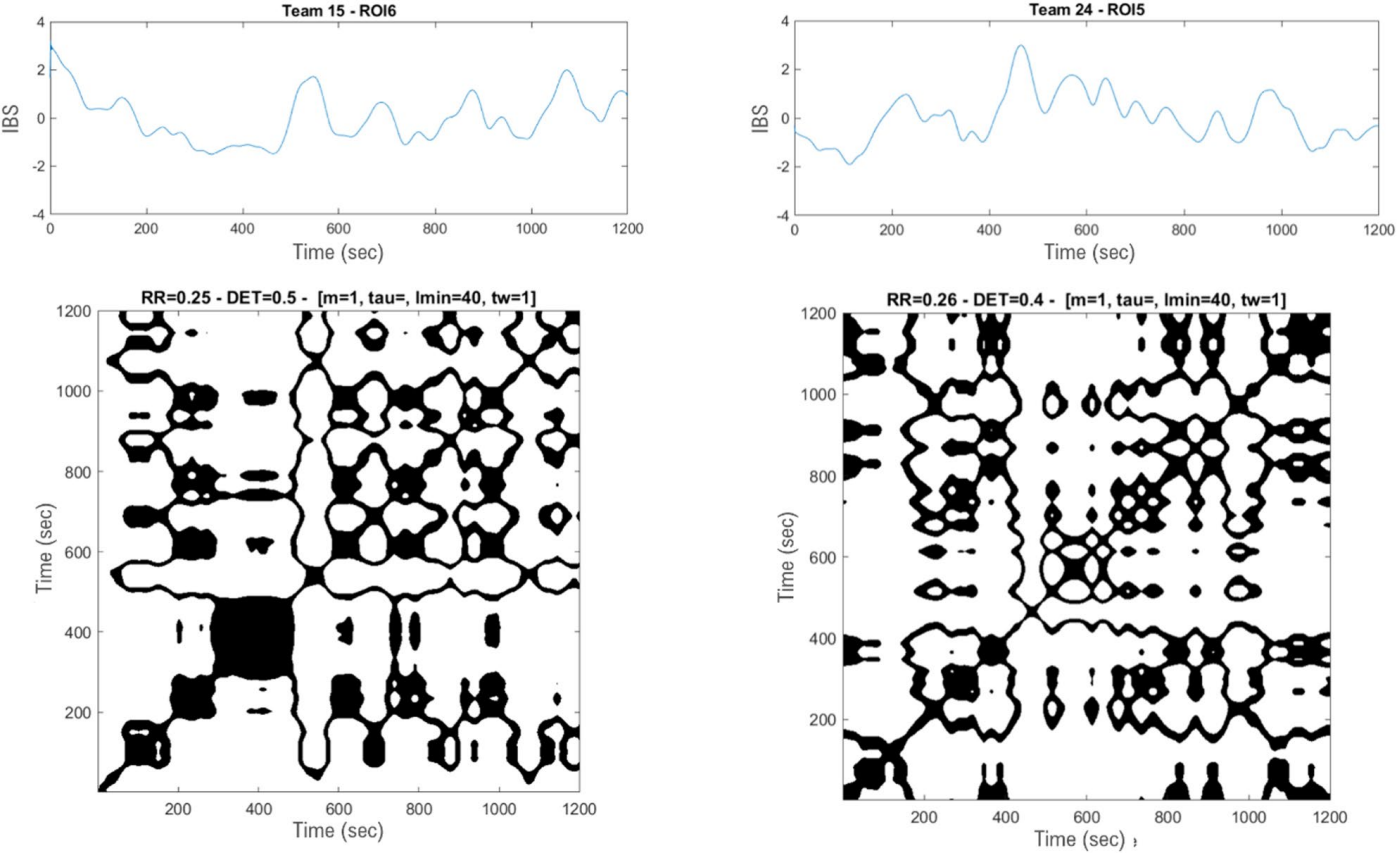
Examples of recurrence plots for two dyads. Top: IBS signals in the ROI 1 (Left fp PFC); Bottom: recurrence plots, black dots mark when the signal revisits a similar state, the main black diagonal represents the signal compared to itself

The third feature, the temporal regularity of IBS signals, was quantified using the %DET. The %DET (% determinism) is a feature derived from recurrence quantification analysis (RQA), a non-linear method for analyzing the structure of time-series data [79], already used to examine the temporal regularity of time series produced by teams [49, 62]. RQA generates recurrence plots (RPs), where the time series is plotted against itself along the x- and y-axes. A dot is marked whenever the system revisits a similar state, resulting in a visual map of the signal’s temporal structure. The main diagonal, which represents the signal compared to itself, is always fully marked [31, 121]. The spatial organization of dots on the RP reveals signal regularities: while random signals display isolated and scattered dots, structured signals produce coherent diagonal patterns. The %DET is the proportion of recurrence points forming diagonal lines, relative to the total number of recurrence points, and serves as an indicator of how temporally structured the signal is. A higher %DET reflects a greater degree of regularity in the IBS signal over time. To compute %DET, IBS signals were first reconstructed in phase space using the MATLAB function “phasespace1”, with the embedding dimension set to *m = 1*. Recurrence analysis was then performed using the “crqa” function from the *CRPtoolbox*. As recurrence analysis parameters need to be adapted to the data and to the objectives of the analysis [79], they were selected following a systematic exploration of parameter ranges. This exploration aimed to avoid floor and ceiling effects and to maximize the variability and sensitivity of recurrence rate (RR) and %DET measures (see Supplementary Material 4). Based on this procedure, the embedding dimension (m) was set to 1 to capture the slow temporal dynamics of the fNIRS signals; the radius (*ε*) was computed individually for each signal using the “radsel” function [87]; the minimum diagonal line length (*lmin*) was set to 40; and the Theiler window to 1. Examples of recurrence plots are available in Fig. 4.

To ensure that %DET values were comparable across dyads within each ROI, recurrence rate (RR) histograms were inspected and the absence of substantial variation in RR across dyads was checked (see Supplementary Material 4).

### Communication measurement and analysis

#### Speech recording and diarization

Each participant’s voice was captured using a clip-on microphone connected to an H6 recorder (Zoom), with a sampling rate set at 4400 Hz, resulting in two audio tracks, one per participant. Voice data processing was performed using Python software, using the *pyannote.audio* library for speaker diarization [15, 23]. The audio track of each participant was processed with a pre-trained model for speaker diarization (Pipeline.from_pretrained “pyannote/speaker-diarization-3.1”) to extract the time segments containing the participant’s speech. These speech segments were then converted into binary time series sampled at 10 Hz, where 1 indicates that the participant was speaking, and 0 indicates silence.

#### Communication features quantification

The effect of the disruption was analyzed in terms of two dimensions of communication. First, the *total speaking time* of the dyad, which reflects the overall engagement in verbal interaction, was defined as the sum of the speaking durations of both the Guide and the Drawer. Second, the distribution of speaking time among team members, captured by the dyad *speech ratio*, provides insight into the balance of communication within the dyad. This was calculated by dividing the speaking time of the Guide by that of the Drawer. Due to the nature of the task, in which the Guide provided verbal instructions, speech ratios greater than 1 were expected. A ratio close to 1 would indicate balanced participation between the two participants, whereas higher values would reflect increasing asymmetry, with the Guide speaking for longer than the Drawer.

Both communication features were computed before and after the 10-minute event.

### Performance measurement

**Fig. 5:**
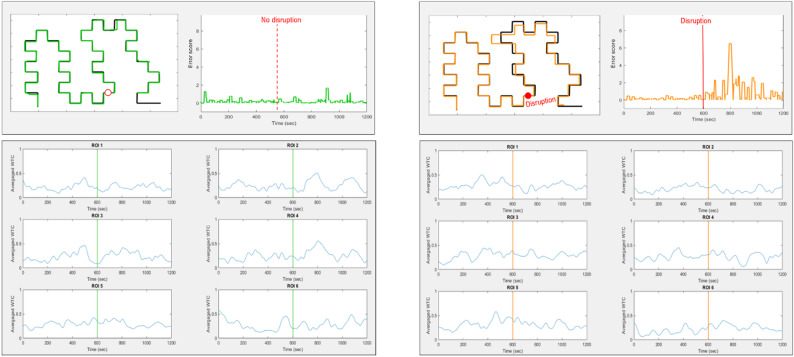
Examples of team performance. Left panels: one dyad in the control group: top left: realization and the error score over time, bottom left: IBS signals in the 6 ROIs. Right panels: one dyad in the experimental group: top left: realization and the error score over time, bottom left: IBS signals in the 6 ROIs

Team performance was assessed based on the accuracy of the drawn path, defined as the distance between the trajectory produced by the dyad and the reference path to be replicated. To compute this distance, the coordinates of the moving cursor were recorded at a sampling rate of 10 Hz throughout the task. For each point on the dyad’s path, a local error was calculated as the Euclidean distance between that point and the nearest point on the reference path. A local error value of zero indicates perfect alignment with the reference path, whereas larger values reflect greater deviation. The overall task performance score was obtained by summing all local error values across the trajectory (see Fig. 5).

### Subjective perception of task difficulty

After completing the task, participants were asked to independently rate their subjective perception of task difficulty for both the first and second halves of the task. They did so by placing a mark on a 10-cm visual analog scale (VAS) without units. The perceived difficulty score (out of 10) was determined by measuring the distance in centimeters between the left end of the scale (representing minimum difficulty) and the participant’s mark.

### Statistical analyses

All statistical analyses were conducted with R [24]. We used linear mixed-effects models (LMMs) to account for the nested structure of our data, in which repeated measurements across the six ROIs are nested within dyads. By including ROI as a random effect, LMMs enable hypothesis testng across all ROIs while appropriately accounting for the non-independence of observations. This approach is recommended in neuroscience research to reduce the risk of Type I errors in hierarchical and repeated-measures designs [125]. LMMs were implemented using the {lme4} package for model comparison, and the {lmerTest} package [68] was used to obtain significance tests for the fixed effects.

#### Control analyses

Before testing whether the disruption led to changes in IBS, methodological precautions were taken to ensure that: (1) the teamwork interaction induced IBS; (2) the disruption effectively increased task difficulty, leading to a significant drop in team performance; and (3) the disruption indeed altered the teamwork interaction at the behavioral level, with a significant change in team communication. These three checks were conducted to ensure the effectiveness of the experimental manipulation.

##### IBS during teamwork

The first control analysis aimed to ensure that the observed IBS within dyads was not spurious, but truly related to social interaction. In line with recent recommendations in the hyperscanning literature [57, 88, 96], we used permuted dyads as a control condition, as this approach is considered more appropriate and ecologically valid than comparing task activity to a resting-state baseline. Therefore, we compared the IBS of the real dyads with the IBS of permuted dyads. While real dyads were composed of individuals who had actually interacted with each other during the experimental team task, the “permuted dyads” were constructed by pairing Guides and Drawers who had not interacted, creating all possible non-interacting combinations, as in Reindl et al. [103]. This comparison was based on data from the 10-minute period preceding the disruption. Differences in IBS distributions were tested using an ANOVA on linear mixed-effects models. Two models were constructed and compared. The first model was a full model that included, as a fixed effect, the variable representing dyad type (real or permuted, referred to as *DyadType*). The second model was a null model that did not include dyad type as a fixed effect. Since the data from the six ROIs within the same dyad are not independent, we accounted for the nested structure of the data (ROIs nested within dyads) by including ROIs as a random effect. Models were fitted using restricted maximum likelihood to ensure comparability. The model formulas are provided in Table 1.

**Table 1:** Specification of the linear mixed-effects models used in the current study ({lmer} package in R)

Model	Dependent variable	Fixed effect	RE	Formula
Control analyses				
Full	IBS level	Dyad Type (real/permuted)	ROI	IBS ~ DyadType + (1|ROI)
Null	IBS level	None	ROI	IBS ~ 1 + (1|ROI)
Full	Errors score	Time x Group	Dyad	ErrorScore ~ Time x Group + (1|Dyad)
Reduced	Errors score	Time + Group	Dyad	ErrorScore ~ Time + Group + (1|Dyad)
Full	Perceived difficulty score	Time x Group	Individual	PerceivedDifficulty ~ Time x Group + (1|Individual)
Reduced	Perceived difficulty score	Time + Group	Individual	PerceivedDifficulty ~ Time + Group + (1|Individual)
Full	Total speaking time	Time x Group	Dyad	Total SpeakingTime ~ Time x Group + (1|Dyad)
Reduced	Total speaking time	Time + Group	Dyad	Total SpeakingTime ~ Time + Group + (1|Dyad)
Full	Speech ratio	Time x Group	Dyad	SpeechRatio ~ Time x Group + (1|Dyad)
Reduced	Speech ratio	Time + Group	Dyad	SpeechRatio ~ Time + Group + (1|Dyad)
Hypotheses testing (IBS)				
Full	IBS level	Time x Group	Dyad/ROI	IBSlevel ~ Time x Group + (1| Dyad/ROI)
Reduced	IBS level	Time + Group	Dyad/ROI	IBSlevel ~ Time + Group + (1| Dyad/ROI)
Full	IBS slope	Time x Group	Dyad/ROI	IBSslope ~ Time x Group + (1| Dyad/ROI)
Reduced	IBS slope	Time + Group	Dyad/ROI	IBSslope ~ Time + Group + (1| Dyad/ROI)
Full	IBS %DET	Time x Group	Dyad/ROI	IBS%DET ~ Time x Group + (1| Dyad/ROI)
Reduced	IBS %DET	Time + Group	Dyad/ROI	IBS%DET ~ Time + Group + (1| Dyad/ROI)
Complementary analyses - Moderation				
Full	IBS level	Time x Group x Delta	Dyad and ROI	IBSlevel ~ Time x Group x Delta + (1|Dyad) + (1|ROI)
Reduced	IBS level	Time x Group	Dyad and ROI	IBSlevel ~ Time x Group + (1|Dyad) + (1|ROI)
Full	IBS slope	Time x Group x Delta	Dyad and ROI	IBSslope ~ Time x Group x Delta + (1|Dyad) + (1|ROI)
Reduced	IBS slope	Time x Group	Dyad and ROI	IBSslope ~ Time x Group + (1|Dyad) + (1|ROI)
Full	IBS %DET	Time x Group x Delta	Dyad and ROI	IBS%DET ~ Time x Group x Delta + (1|Dyad) + (1|ROI)
Reduced	IBS %DET	Time x Group	Dyad and ROI	IBS%DET ~ Time x Group + (1|Dyad) + (1|ROI)

RE = Random Effect

#### Experimental manipulation check: disruption increased task difficulty

A second set of analyses was conducted to verify the effectiveness of the experimental manipulation – that is, to ensure that the disruption indeed resulted in a significant increase in task difficulty.

To this end, we first examined whether the *error score* increased after the disruption in the experimental group (i.e., a decrease in performance). We compared two models using an ANOVA. The full model included, as a fixed effect, the interaction term Time × Group; the reduced model included only the main effects of Time and Group, but not their interaction. In both models, the dependent variable was the error score, and a random effect by dyad was included.

We also examined whether participants in the experimental group *perceived the task as significantly more difficult* after the disruption (i.e., perceived task difficulty). Again, we compared two models using an ANOVA. The full model included, as a fixed effect, the interaction term Time × Group; the reduced model included only the main effects of Time and Group, but not their interaction. In both models, the dependent variable was perceived difficulty score, and a random effect by participant was included. The model formulas are provided in Table 1.

#### Experimental manipulation check: disruption led to a change in teamwork interaction

A third set of analyses was conducted to verify the effectiveness of the experimental manipulation – that is, to ensure that the disruption indeed resulted in a change in teamwork social interaction.

To this end, we examined whether the *total speaking time* changed after the disruption in the experimental group. We compared two models using an ANOVA. The full model included, as a fixed effect, the interaction term Time × Group; the reduced model included only the main effects of Time and Group, but not their interaction. In both models, the dependent variable was the total speaking time (as referred as TotalSpeaking), and a random effect by dyad was included.

We also examined whether the *speech ratio* changed after the disruption in the experimental group. Again, we compared two models using an ANOVA. The full model included, as a fixed effect, the interaction term Time × Group; the reduced model included only the main effects of Time and Group, but not their interaction. In both models, the dependent variable was speech ratio (as referred as SpeechRatio), and a random effect by dyad was included. The model formulas are provided in Table 1.

#### Effect of the disruption on IBS

To test the effect of the disruption on interbrain synchrony (IBS). Three separate ANOVAs were conducted—one for each IBS feature (i.e., level, slope, and %DET). Each ANOVA compared two models: a full model that included the interaction term *Time × Group* as a fixed effect, and a reduced model that included only the main effects of *Time* and *Group*, without the interaction. Dyad and ROI were included as random effects, with ROIs nested within each Dyad to account for the hierarchical structure of the data. The model formulas are provided in Table 1. IBS outcomes were not z-standardized prior to model fitting. Therefore, to facilitate interpretation across IBS outcomes, standardized effect sizes (standardized beta coefficients) and their 95% confidence intervals were computed for the Time × Group interaction.

#### Complementary analyses

According to theoretical models, collective phenomena at the cognitive level (“C”) are thought to emerge through the behavioral interaction (“B”, such as team communication). Therefore, it is plausible that dyads exhibiting larger behavioral adaptation —specifically, changes in team communication – may also experience greater changes in IBS, whereas dyads with more stable communication may have maintained stable IBS levels. To investigate this possibility, we tested whether the magnitude of change in communication moderated the relationship between disruption and IBS. To test this moderating effect, we examined whether the delta in total speaking time and the delta in speech ratio (i.e., the change in these variables following the disruption relative to before) influenced the Time × Group interaction on IBS measures (level, slope, and %DET). We compared a Reduced model including only the Time × Group interaction to a Full model that also includes the three-way interaction term (Time × Group × Delta). The model formulas are provided in Table 1. Similarly to the analyses testing the effects of the disruption on IBS features, we initially attempted to fit models with ROIs nested within dyads, but this specification led to convergence problems, likely due to the limited number of observations per ROI within each dyad. We therefore used the simpler random-effects structure (1 | Dyad) + (1 | ROI) to obtain stable and identifiable models, following standard recommendations (e.g., [10]).

## Results

### Results of the control analyses

#### IBS during teamwork

The ANOVA results on the linear mixed-model analysis revealed that Real dyads (M = 0.27, SD = 0.05) exhibited significantly higher IBS levels than Permuted dyads (M = 0.25, SD = 0.04). Indeed there was a significant fixed effect of Group (χ²(1) = 50.24, *p* < .001). The raw effect size was 0.0183 (95%CI [0.0133, 0.0234]), and the standardized effect size (β) was 0.42 (95%CI [0.30, 0.53]), indicating a medium to large effect (see Table 2).

**Table 2:** Results of the ANOVA on linear mixed models of IBS level before the disruption date, in Real vs. Permuted dyads

Model	npar	AIC	BIC	logLik	χ²	df	*p*	
Reduced	3	-49,154	-49,131	24,580				
Full	4	-49,202	-49,172	24,605	50.24	1	< 0.001	

npar = number of parameters; AIC = Akaike Information Criterion; BIC = Bayesian Information Criterion; logLik = log-likelihood; df = degrees of freedom

#### Experimental manipulation check

Regarding the effect of the disruption on task difficulty, the ANOVA results on the linear mixed models revealed significant Time × Group interaction terms for error score (χ²(1) = 24.99; *p* < .001). The fixed effect of this interaction was significant (β = 0.56, 95%CI [0.37, 0.79], *p* < .001), and explained 43% of the variance in error scores (marginal R² = 0.43). A similar pattern was observed for perceived task difficulty, (χ²(1) = 35.56; *p* < .001), with a fixed effect of the Time × Group interaction (β = 3.01, 95%CI [2.07, 3.96], *p* < .001), explaining 32% of the variance (marginal R² = 0.32). This indicates that, following the disruption, dyads in the experimental group showed a significant increase in both error scores and perceived task difficulty. The detailed results are presented in Table 3.

Regarding the effect of the disruption on team communication, the ANOVA results on the linear mixed models revealed no significant Time × Group interaction terms for total speaking time (χ²(1) = 0.21; *p* = .644). However, we observed a significant Time x Group interaction term for speech ratio (χ²(1) = 8.48; *p* = .04), with a fixed effect (β = 3.17, 95%CI [0.10, 6.25], *p* = .004), explaining 9% of the variance (marginal R² = 0.09). This indicates that while the total speaking time did not change after the disruption in the experimental group, the speech ratio increased (see Table 3). Speaking time thus became less balanced, indicating that the Guide spoke more and/or the Drawer spoke less.

**Table 3:** Results of control analyses: effect of the disruption on team performance and communication

ANOVAs											Summary: M(SD)	
Feature	Model	npar	AIC	BIC	logLik	χ²	df	*p*			Before	After
Errors	Reduced	5	84	97	-37				C		0.41 (0.17)	0.42 (0.24)
	Full	6	61	77	-24	24.99	1	< 0.001	E		0.46 (0.30)	1.05 (0.46)
Perceived difficulty	Reduced	5	889	905	-439				C		4.00 (1.83)	4.20 (1.96)
	Full	6	855	875	-421	35.56	1	< 0.001	E		3.14 (1.69)	6.36 (1.67)
Total speaking time	Reduced	5	1014	1027	-502				C		453 (73)	463 (60)
	Full	6	1016	1031	-502	0.21	1	0.644	E		461 (66)	477 (70)
Speech ratio	Reduced	5	176	189	-83				C		13.11 (12.80)	11.80 (12.89)
	Full	6	169	185	-79	8.48	1	0.004	E		5.68 (3.90)	7.54 (5.04)

npar = number of parameters; AIC = Akaike Information Criterion; BIC = Bayesian Information Criterion; logLik = log-likelihood; df = degrees of freedom; C = control group; E = experimental group; M = mean; SD = standard deviation

### Results of the effect of the disruption on IBS

The three ANOVAs conducted on the linear mixed-effects models revealed no significant Time × Group interaction for any of the three IBS features of interest (level, slope and %DET, see Table 4). While these results provide no evidence for changes in IBS after the disruption, the estimated coefficients and their 95% confidence intervals indicate that small-to-moderate effects cannot be entirely ruled out.

**Table 4:** Results of linear mixed models analyses regarding the effect of the disruption on the IBS features (level, slope and %DET)

ANOVAs									Standardized fixed effect			
Feature	Model	npar	AIC	BIC	logLik	χ²	df	*p*	Coeff	[95%CI]	*p*	pR²
Level	Reduced	6	-1903	-1877	958							
	Full	7	-1902	-1871	958	0.31	1	0.579	-0.085	[-0.388; 0.217]	0.580	0.000
Slope	Reduced	6	-9226	-9200	4619							
	Full	7	-9224	-9194	4619	0.34	1	0.560	0.096	[-0.228; 0.420]	0.561	0.000
DET	Reduced	6	-1167	-1141	589							
	Full	7	-1168	-1138	591	3.32	1	0.068	-0.295	[-0.613; 0.023]	0.069	0.005

npar = number of parameters; AIC = Akaike Information Criterion; BIC = Bayesian Information Criterion; logLik = log-likelihood; df = degrees of freedom; 95%CI = 95% confidence intervals; pR² = partial R²

To further contextualize these findings, simulation-based sensitivity analyses were conducted (see **Supplementary Material 5**). The results indicate that the Time × Group interaction coefficients observed for our data were substantially smaller than the detection thresholds, suggesting that either the disruption has no detectable effect on the three IBS features, or that any existing effect is small. In any case, the presence of small effects cannot be entirely excluded.

### Results of the complementary analyses

Results from the moderation analyses examining whether the magnitude of team communication adaptation could account for differences in IBS change across dyads indicated that including the Delta term did not meaningfully improve model fit. Specifically, both AIC and BIC values showed minimal or no improvement, and the interaction term was not statistically significant for IBS level, as confirmed by the non-significance of the three fixed effects (see Table 5). These findings suggest that changes in team communication did not significantly moderate the effect of disruption on any of the IBS features.

**Table 5:** Results of linear mixed models analyses regarding the moderator effect of change in speech ratio on the disruption–IBS link

Feature	ANOVAs								Fixed effect			
	Model	npar	AIC	BIC	logLik	χ²	df	*p*	Coeff	[95%CI]	*p*	mR²
IBS level	Null	7	-1804	-1774	909							
	Full	11	-1797	-1750	910	1.56	4	0.816	0.001	[-0.002; 0.005]	0.415	0.05
IBS slope	Null	7	-8808	-8778	4411							
	Full	11	-8807	-8760	4415	6.97	4	0.137	0.000	[0.000; 0.000]	0.677	0.01
IBS DET	Null	7	-1121	-1091	568							
	Full	11	-1115	-1068	569	2.24	4	0.692	0.000	[-0.006; 0.006]	0.993	0.01

npar = number of parameters; AIC = Akaike Information Criterion; BIC = Bayesian Information Criterion; logLik = log-likelihood; df = degrees of freedom; 95%CI = 95% confidence intervals; mR² = marginal R²

## Discussion

The present study aimed to examine whether IBS could detect a disruption in teamwork, by investigating the effect of a sudden and unpredictable increase in task difficulty on IBS dynamics.

Preliminary control analyses suggest that the experimental manipulation was effective in both inducing IBS and disrupting the teamwork interaction. Specifically, IBS was found to emerge between participants during the current teamwork interaction, as real dyads exhibiting significantly higher IBS levels compared to permuted dyads. The disruption also impacted team performance, as evidenced by an increase in error scores in the experimental group, along with a clear rise in perceived task difficulty. While total speaking time remained unchanged, the disruption led to a significant shift in the speech ratio between the Guide and the Drawer, indicating an adaptation in communication dynamics. Taken together, these results provide support for the effectiveness of the experimental manipulation. Regarding potential changes in IBS, the analyses did not reveal robust evidence for disruption-related differences across the three IBS features examined, including overall IBS level, IBS trajectory over time (slope), and temporal regularity (%DET).

The absence of clear evidence of neural synchrony change observed in the present study is consistent with previous findings on cardiac and electrodermal synchronies, that was found to be resistant to a sustained disruption consisting in a computer malfunction [52]. This result suggests that IBS – similarly to cardiac and electrodermal synchronies – does effectively not change, raising the possibility that these physiological team level phenomena may possibly reflect enduring aspects of interpersonal interaction that remains stable after a disruption.

When taken as a whole, the results of the present study also reveal several important observations. Teams were clearly affected by the disruption, as indicated by both behavioral and subjective evidence: their performance declined, and participants reported greater perceived difficulty. In response, teams adapted by modifying the way they interacted, particularly through changes in their communication patterns. Despite these challenges, they were able to sustain effective cooperation and continue working together on the task. Interestingly, no reliable changes in IBS were detected following the disruption. Rather than being interpreted as a null result, this pattern may suggest that effective teamwork was maintained despite the disruption. If IBS remains stable while individuals continued to work together – albeit in a different manner – this would suggest that IBS may primarily indicate *whether* individuals are engaged in teamwork, rather than *how* they interact while doing so. Indeed, teams may have adapted very rapidly to the disruption, allowing them to maintain both IBS and collaboration. Given that participants had already been working together for ten minutes before the disruption occurred, such adaptation may have taken place quickly enough to result in no observable change in IBS.

The lack of robust evidence for IBS change observed in this study also challenges the notion that team emergent states are necessarily modified following a disruption. More broadly, it raises questions about the extent to which IBS can directly reflect team cognition, as previously suggested [35, 110]. This finding relates to the broader issue of the social and cognitive significance of IBS – a central topic in current theoretical debates within the hyperscanning literature [51, 55, 57, 97]. Clarifying whether IBS can serve as a measure of team cognition will require systematic research using a multi-source approach that combines IBS measures with subjective assessments of team members’ mental representations [89], in order to clarify whether IBS truly reflects team-level cognitive constructs such as shared mental models [35]. More generally, clarifying the functional interpretation of IBS requires linking IBS measures to subjective assessments of team-level phenomena. Although several psychological interpretations of IBS have been proposed, there is currently no clear consensus regarding its psychological meaning (e.g., [51, 55]). Establishing the construct validity of IBS requires systematic examination of its relationship with subjective measures, such as perceived teamwork, team cohesion, or interpersonal closeness (Réveillé et al. [104, 105]). Incorporating brief post-task questionnaires (e.g., [36, 72]) would help future studies clarify which aspects of team functioning IBS actually reflects.

The results of this study, which suggest no changes across the three IBS features (level, slope, and %DET) following the disruption, may also suggest that IBS was affected by the teamwork disturbance, but that the methodology employed here was not sensitive enough to detect it. In this case, the present findings offer an initial step toward understanding how team adaptation manifests at the neurophysiological level and how it might best be captured methodologically. Three methodological aspects warrant further discussion. The first aspect concerns the link between the nature of the disruption and the adaptation process. The disruption proposed in the current study, which effectively increased task difficulty, seems to have primarily affected task-related behavioral processes – team communication and performance – rather than the interpersonal phenomena captured by IBS. Notably, external disruptions – such as changes in task demands or unexpected events like the one introduced here – are generally considered less detrimental to team adaptation than internal disruptions [21]. Indeed, external disruptions primarily require adjustments related to the task itself and may only secondarily influence interpersonal interactions. In contrast, internal disruptions (e.g., replacing a team member or altering communication capacities between members) directly interfere with interpersonal mechanisms, producing stronger perturbations in team dynamics. Thus, teams facing external disruptions can often continue their task by relying on pre-established interaction patterns and routines – an option that is not available when the disruption originates within the team itself. Consistent with this distinction, previous hyperscanning studies have reported IBS changes when disruptions increased both workload and cooperation demands [5, 119], which represent disruptions internal to the team. Our results may suggest that the interpersonal processes captured by IBS are not sensitive to external disruptions of low intensity.

A second aspect concerns the fNIRS measurement itself, which may partly account for the absence of clear IBS changes following the disruption in the experimental group. It is well established that fNIRS signals recorded over the prefrontal cortex are influenced by superficial physiological signals, such as scalp and skin blood flow (e.g., [116]). Although a PCA-based correction was applied to attenuate global systemic noise, and IBS was quantified within a low-frequency band commonly associated with cortical hemodynamic activity in order to reduce physiological artifacts, residual extracerebral contributions cannot be entirely ruled out. One possible interpretation is that IBS features partly reflected shared systemic signals in the current study. As a result, the sensitivity to detect disruption-related changes in the experimental group may have been reduced. Future studies investigating disruption-related changes in IBS would particularly benefit from incorporating short-separation channels to reduce ambiguity regarding the contribution of superficial hemodynamics [88, 126]. Another possible reason for the absence of IBS changes is variability related to speech and movement. Unlike EEG, fNIRS does not directly capture facial muscle activity; however, facial expressions may indirectly affect fNIRS signals through associated head movements and changes in optode positioning [7, 102]. While these effects were addressed using established motion correction procedures [90, 126], residual artifacts may persist, particularly around discrete task events such as the disruption. Consequently, changes in verbal and non-verbal behavior may have introduced additional variability across dyads, potentially masking subtle IBS changes in the experimental group. Finally, state-level factors may also have influenced IBS. During prolonged collaborative tasks, fatigue, alertness, and arousal are known to affect both behavior and neural measures, including fNIRS signals [29, 124]. Although the inclusion of a control group allowed us to account for time-related effects common to both groups, it remains possible that mental fatigue or reduced arousal affected both groups similarly, interacted with the disruption, and consequently attenuated potential IBS changes. Future studies would benefit from incorporating subjective ratings (e.g., perceived fatigue or workload) and physiological indicators (e.g., heart rate, pupil diameter, [76, 99]) to better characterize the contribution of psychophysiological states to IBS dynamics, particularly in the context of discrete task disruptions.

A third aspect concerns the broader question of how IBS adaptations can be most effectively captured. The methodological choices adopted in the present study – particularly the selection of brain regions of interest, the computation of IBS in homotopic areas, and the use of WTC – were guided by current knowledge on IBS and existing recommendations in fNIRS hyperscanning research [25, 51, 96, 103]. At the same time, the field continues to offer a wide range of methodological options for assessing IBS – especially regarding brain region selection, connectivity measures, inter-brain mapping strategies, and the appropriate frequency band. Recent findings indicate that these analytical choices can substantially influence IBS outcomes [129]. This highlights the broader need to identify the most appropriate approach for quantifying IBS when the goal is to detect subtle, within-task changes. Achieving this will require systematic comparisons of existing IBS quantification techniques, in order to clarify the scope and limitations of each method in capturing IBS dynamics, and to provide researchers with objective evidence to guide informed methodological decisions aligned with their research questions [129]. More specifically, in the present task, temporal delays between one participant’s behavior and the other’s response suggest that lagged IBS analyses could provide complementary insights. However, the choice of temporal lag is a critical methodological issue that can substantially influence results, and no clear consensus currently exists regarding which lag should be used [78]. As such, lagged synchrony analyses represent an important direction for future methodological investigations.

Continuing the reflection on how IBS changes may be captured, our findings also raise questions about how adaptation in IBS might manifest. It is possible that changes in IBS occur along dimensions other than the features investigated here. Our behavioral findings provide a relevant illustration: while some aspects of communication (e.g., speech ratio) were sensitive to the disruption, others (e.g., total speaking time) remained stable. This pattern suggests that team adaptation may selectively influence certain dimensions of an emergent state without altering the construct as a whole. Applied to IBS, this reasoning implies that the disruption could have affected other, unexamined dynamic aspects of IBS beyond those commonly described in the teamwork dynamics literature – namely, overall level, developmental trajectory, and recurrent patterns. Previous studies have, for instance, demonstrated that changes in emergent states can be detected using measures such as entropy (e.g., [111]) or the Lyapunov exponent (e.g., [43]), both are indicators of the temporal dynamics of IBS, which could be modified following a disruption. Consequently, sophisticated dynamic analyses could further extend our understanding of IBS by revealing subtle changes not observable with the current level of analysis.

Similarly, existing literature on team adaptation – including empirical evidence – suggests that a sustained disruption can induce a lasting shift in team state, from an initial state A to a new state B. Since our results did not reveal such long-term changes in IBS, we also explored potential short-term variations by examining IBS dynamics around the time of the disruption (see Supplementary Material 6). The absence of significant effects in these additional analyses leads us to consider the possibility of transient effects that could be captured through alternative analytical approaches, such as sliding-window analyses (e.g., Grimm et al. [45]). In this regard, theoretical frameworks conceptualizing teams as nonlinear dynamic systems (e.g., Arrow et al. [3]; [44, 86]) along with corresponding nonlinear analytical methods and features [50], may provide appropriate tools for identifying the dynamic transitions underlying team adaptation.

Pursuing this reflection on the temporal nature of IBS dynamics also invites consideration of the physiological signals used to capture such changes. If adaptation-related effects are short-lived or transient, they may not be adequately captured by the low-frequency components of the fNIRS signal associated with the hemodynamic response. Combining fNIRS with faster-changing physiological measures, such as cardiac or respiratory activity, may therefore help capture transient synchronization changes during team adaptation [14].

The present study has several methodological limitations that should be acknowledged. First, as discussed above, several unmeasured factors may have influenced the fNIRS signals and the resulting IBS features. The absence of short-separation channels, the lack of direct measures of arousal and fatigue, and speech-related movements limited our ability to fully control for systemic physiological effects in the fNIRS signals. In addition, participant characteristics known to influence fNIRS signal properties – such as skin pigmentation, hair properties, and handedness – were not systematically assessed, whereas melanin content can affect near-infrared light penetration (e.g., skin pigmentation, hair properties, Yücel et al. [126]), and handedness may influence hemispheric activation [4, 22]. Second, regarding IBS quantification, although methodological choices were made in accordance with current fNIRS hyperscanning guidelines [96, 103], measuring IBS exclusively between homotopic brain regions and relying on undirected synchrony metrics may not fully capture the complexity of information exchange in an asymmetric teamwork task. Alternative approaches, such as directed or lagged analyses, may provide complementary insights in future work. Third, several individual variables that could influence interaction dynamics and task performance were not controlled for. Participants’ visual and auditory status was assessed via self-report but not objectively measured, and prior experience with video games or teamwork was not evaluated. Given that such factors may influence adaptability to task demands and disruptions [69], their absence prevented us from accounting for potential confounding effects on both behavioral and neurophysiological outcomes. In addition, the experimental and control groups could not be considered fully equivalent with respect to dyad gender composition (see Supplementary Material 1). Although equivalence was observed for male–female dyads, differences in other gender composition categories may have influenced communication patterns and IBS. Finally, communication was operationalized using total speaking time and speech ratio. While these measures capture important aspects of interaction, other dimensions of communication—such as lexical or semantic content [33, 113] - as well as additional behavioral markers, including unintentional motor synchrony [106], may have been sensitive to disruption-related changes that were not detected by the present measures. Future studies adopting a more fine-grained multimodal approach may help further elucidate how disruptions affect teamwork dynamics and interbrain synchrony.

## Conclusion

As a conclusion, we consider this study as a preliminary investigation showing that, as measured by fNIRS hyperscanning in the prefrontal cortex and temporoparietal junction using WTC, no reliable disruption-related changes in IBS were detected. However, before drawing definitive conclusions about the insensitivity of IBS to teamwork disruptions, future studies investigating the effects of various disruptions on more complex aspects of IBS dynamics appear necessary. Moreover, this study points to the broader challenge of methodological diversity in IBS quantification and underscores the need to better understand what each approach captures, in order to support methodological decisions in hyperscanning.

## Supplementary Information

Below is the link to the electronic supplementary material.

Supplementary Material 1

## Data Availability

The datasets used and/or analyzed during the current study are available from the corresponding author on reasonable request.
