# Supplementary material for: Investigating interbrain synchrony under teamwork disruption: an fNIRS hyperscanning study
Source: Behav Brain Funct. 2026 Feb 8;22:12. doi: 10.1186/s12993-026-00320-6 (PMC12977408; doi:10.1186/s12993-026-00320-6)
Supplement: Supplementary file 1 — Supplementary Material 1 [file 12993_2026_320_MOESM1_ESM.docx]

**SUPPLEMENTARY MATERIALS**

# SM 1. Description of the sample

- **Description of the sample**

**Table SM-1.1:** Description of the sample

|  | **Individuals** | | | |  | **Dyads** | | | |
| --- | --- | --- | --- | --- | --- | --- | --- | --- | --- |
|  | **Age: M (SD)** |  | **Gender: Nb (%)** | |  | **Gender: Nb (%)** | | | |
|  |  |  | **Female** | **Male** |  | **FF** | **FM** | **MF** | **MM** |
| **All sample** | 22.75 (3.22) |  | 69 (65.1%) | 37 (34.9%) |  | 27 (50.9%) | 9 (17%) | 6 (11.3%) | 11 (20.8%) |
| **C** | 23.04 (3.77) |  | 38 (76%) | 12 (24%) |  | 16 (64%) | 3 (12%) | 3 (12%) | 3 (12%) |
| **E** | 22.5 (2.67) |  | 31 (55.4%) | 25 (44.6%) |  | 11 (39.3%) | 6 (21.4%) | 3 (10.7%) | 8 (28.6%) |
| Note: C = Control Group; E = Experimental Group; M = Mean; SD = Standard Deviation; Nb = count; % = percentage; **FF** = team composed of two women; **MM** = team composed of two men; **FM** = team with a female Guide and a male Drawer; **MF** = team with a male Guide and a female Drawer | | | | | | | | | |

- **Test of the gender composition equivalence between Control and Experimental group**
- **Rational and analyses**

Considering the differences in dyad gender composition between Control and Experimental group, we first assessed whether these differences were statistically detectable using a chi-square test. However, because a non-significant chi-square result does not imply the absence of differences, we also conducted additional equivalence testing using the Two One-Sided Tests (TOST) procedure to test whether existing differences were sufficiently small to be considered negligible.

- **Results**

Results from the chi-square indicate no detectable difference in dyad gender composition between the control and experimental groups (χ²(3) = 4.04, p = 0.26, see **Table SM-1.2**)

**Table SM-1.2:** Contingency table observed and expected frequencies

|  |  | **Dyads** | | | |
| --- | --- | --- | --- | --- | --- |
| **Group** | **Type** | **FF** | **FM** | **MF** | **MM** |
| **C** | **Observed** | 16 | 3 | 3 | 3 |
| **C** | **Expected** | 13 | 5 | 3 | 5 |
| **E** | **Observed** | 11 | 6 | 3 | 8 |
| **E** | **Expected** | 14 | 5 | 3 | 6 |
| Note: **FF** = team composed of two women; **MM** = team composed of two men; **FM** = team with a female Guide and a male Drawer; **MF** = team with a male Guide and a female Drawer | | | | | |

Results from the TOST procedure indicate that the equivalence criterion was met only for the male-female (MF) category, indicating that the dyad gender composition of the Experimental and Control groups cannot be considered fully equivalent (see **Table SM-1.3**).

**Table SM-1.3:** Results of the Two One-Sided Tests (TOST) procedure

| **Dyads** | **pE** | **pC** | **difference**  **pE - pC** | **CI_low** | **CI_high** | **pL** | **pU** | **p** |
| --- | --- | --- | --- | --- | --- | --- | --- | --- |
| **FF** | 0.39 | 0.64 | -0.25 | -0.47 | -0.03 | 0.491 | 0 | 0.491 |
| **FM** | 0.21 | 0.12 | 0.09 | -0.07 | 0.26 | 0 | 0.062 | 0.062 |
| **MF** | 0.11 | 0.12 | -0.01 | -0.16 | 0.13 | 0.003 | 0.001 | **0.003** |
| **MM** | 0.29 | 0.12 | 0.17 | -0.01 | 0.34 | 0 | 0.216 | 0.216 |
| Note: **pE** = proportion in the experimental group; **pC** = proportion in the control group; **CI** = 95% confidence interval for the difference in proportions (pE − pC); **pL** = p-values of the lower- bound and **pU** = p-values of the upper-bound. | | | | | | | | |

# SM 2. Participants and data flow chart


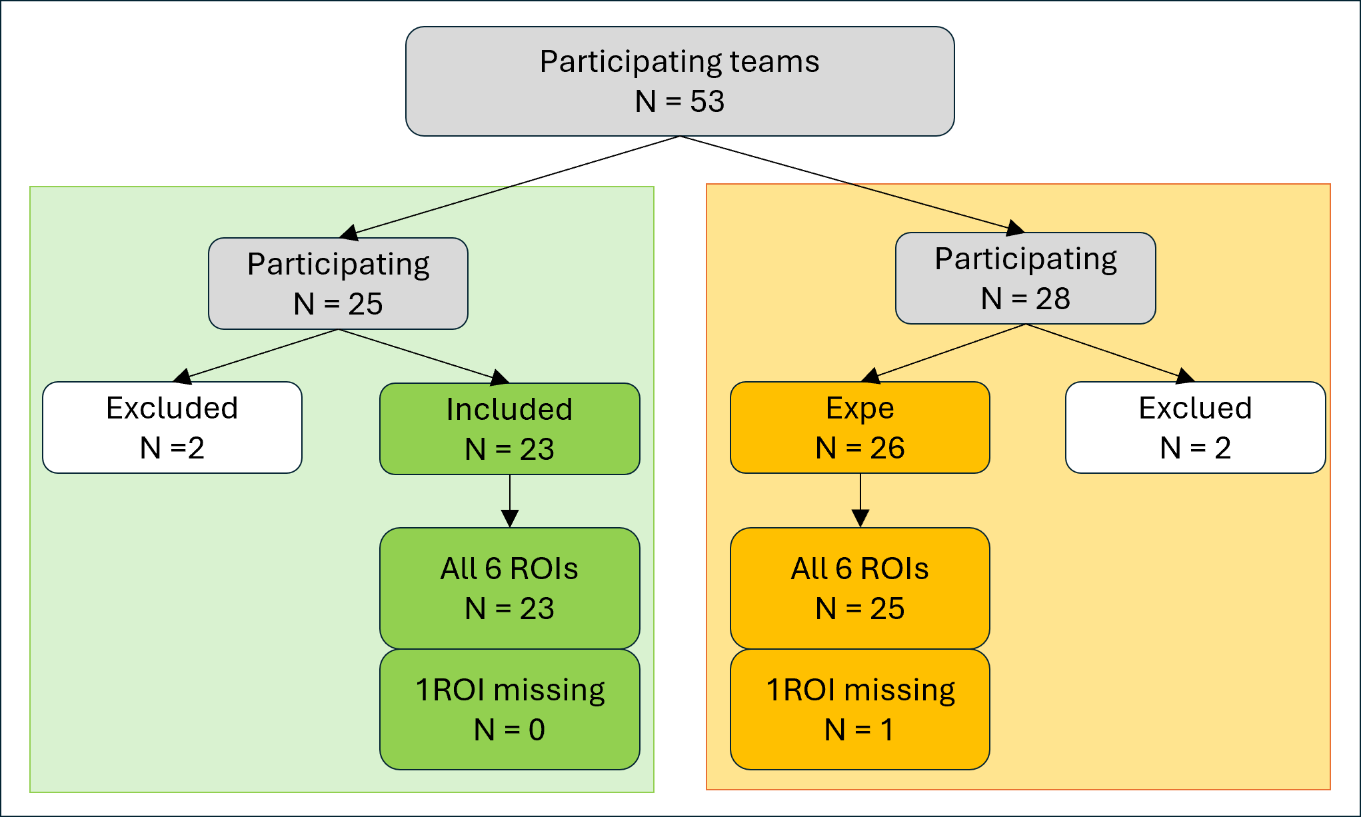


**Figure SM-2:** Participant Flowchart

# SM 3. Analyses with deoxyhemoglobin (HHb)

- **Results**

**Table SM-3:** Results of the Linear Mixed Models: effect of disruption on IBS (level, slope and %DET) for deoxyhaemoglobin (HHb)

|  | **ANOVA** | | | | | | |  | **Fixed effect** | | | |  | **Summary:** | |
| --- | --- | --- | --- | --- | --- | --- | --- | --- | --- | --- | --- | --- | --- | --- | --- |
| **Model** | **npar** | **AIC** | **BIC** | **logLik** | **χ²** | **df** | **p** |  | **Coeff** | **[95% IC]** | **p** | **mR²** |  | **M(SD)** | |
| Full | 3 | -46679 | -46657 | 23343 |  |  |  |  | 0.004 | [-0.002; 0.009] | 0.173 | 0 |  | **Real** | 0.26 (0.05) |
| Null | 4 | -46679 | -46649 | 23344 | 1.86 | 1 | 0.173 |  |  |  |  |  |  | **Perm** | 0.26 (0.05) |

Note: npar = number of parameters; AIC = Akaike Information Criterion; BIC = Bayesian Information Criterion; logLik = log-likelihood; df = degrees of freedom; mR² = marginal R²;M = mean; SD = standard deviation

- **Interpretation**

Since IBS was not higher in real dyads compared to permuted dyads in HHb data, subsequent analyses (i.e., examining the effect of the disruption on IBS) were not conducted.

# SM 4. Ensuring the comparability of %DET by verifying recurrence rates (RR)


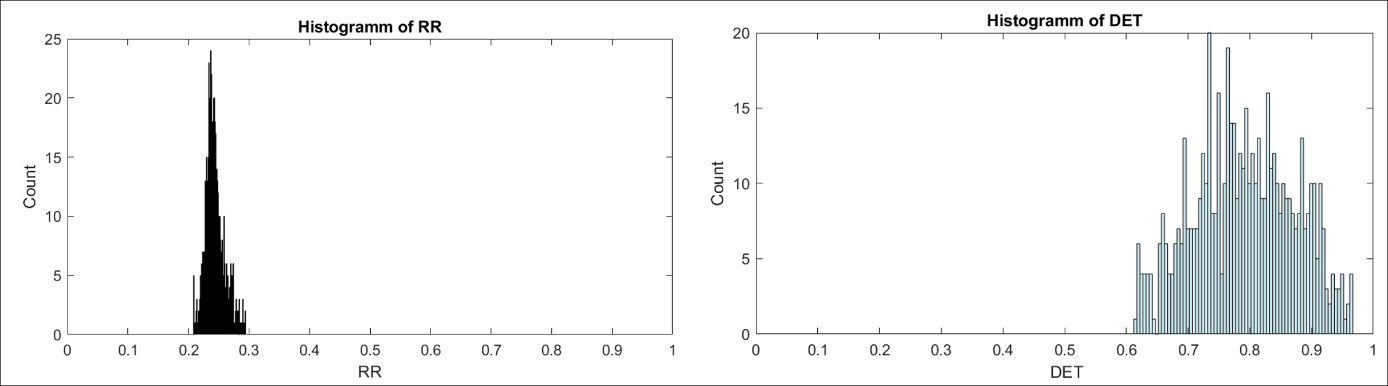


**Figure SM-4**: Histograms of RR and %DET of all teams mixed, all ROIs mixed, and all time mixed (before and after the disruption date)

**Table SM-4:** Linear Mixed Model and summary of findings on recurrence rate (RR)

| **ANOVA** | | | | | | | | |  | **Fixed effect** | | | |
| --- | --- | --- | --- | --- | --- | --- | --- | --- | --- | --- | --- | --- | --- |
| **Feature** | **Model** | **npar** | **AIC** | **BIC** | **logLik** | **Chisq** | **df** | **p** |  | **Coeff** | **[95% IC]** | **p** | **mR²** |
| **RR** | Null | 6 | -2972 | -2945 | 1492 |  |  |  |  |  |  |  |  |
|  | Reduced | 7 | -2970 | -2939 | 1492 | 0.13 | 1 | 0.721 |  | -0.001 | [0.007; 0.005] | 0.723 | 0.01 |

Note: npar = number of parameters; AIC = Akaike Information Criterion; BIC = Bayesian Information Criterion; logLik = log-likelihood; df = degrees of freedom; mR² = marginal R²

# SM 5. Sensitivity analyses

- **Rationale**

The main analyses indicated an absence of significant time × group effects on the IBS features. To further interpret these non-significant findings, we conducted simulation-based sensitivity analyses to determine the minimum interaction effect size that the present study was able to detect. The goal was to assess whether the non-significant results reflected a genuine absence of effects, or whether the possible presence of very small effects (below the sensitivity) of the study should be considered.

- **Methods**

First, we refitted the same LMM as in the main analysis (including Time, Group, and their interaction as fixed effects, and random intercepts for ROI nested within dyads), in order to examine the observed power associated with the estimated Time × Group interaction for the three IBS features (level, slope, and %DET). Given the very small estimated interaction coefficients (ꞵ_observed), the corresponding observed powers were necessarily low (see Table SM-5). These low observed power estimates served to motivate subsequent simulation-based sensitivity analyses.

Second, we aimed to quantify the smallest Time × Group interaction effect that the present design could reliably detect (ꞵ_detectable_β). To this end, we conducted a simulation-based sensitivity analysis.

Specifically, we systematically tested different assumed effect sizes (β) of the Time × Group interaction in the fitted model. For each assumed β, multiple simulated datasets were generated the Time × Group interaction was tested in each. Statistical significance was assessed at α = .05, and adequate statistical power was defined a priori as 80%. This procedure enabled us to ascertain the smallest Time × Group interaction coefficient (β) that the present design could detect in at least 80% of simulations.

- **Results**

**Table SM-5**: Results of the sensitivity analyses

| IBS feature | ꞵ_observed | Power_observed | ꞵ_detectable |
| --- | --- | --- | --- |
| Level | −0.004 | 0.114 | 0.020 |
| Slope | 7.67 x 10^−6^ | 0.118 | 4.00 x 10^−4^ |
| %DET | −0.026 | 0.488 | 0.040 |

Simulation-based sensitivity analyses indicated that the present design had sufficient power (80%) to detect Time × Group interaction effects of approximately ±0.020 for Level, ±4.00 x 10^−4^ for Slope, and ±0.040 for %DET. The Time × Group interaction coefficients observed in our data were substantially smaller than these detection thresholds, suggesting that either no interaction effect is present, or that any existing effect is necessarily small in magnitude. In any case, the presence of effects smaller than these detection thresholds cannot be entirely excluded.

# SM 6. Complementary analysis: local effect of the disruption on IBS features

- **Rationale**

In this study, we drew upon existing literature on team adaptation—particularly empirical work suggesting that sustained disruptions can induce a lasting shift in team state, from an initial configuration (state A) to a new, reorganized one (state B). Guided by this perspective, we focused on detecting durable changes in IBS features, but found no long-term effects in our data. However, it is also possible that a sustained disruption produces only transient effects, specifically during the period of transition between state A and state B. This possibility aligns with the two-phase model of team adaptation (Hale et al., 2016; Summers et al., 2012), which distinguishes between a disruption phase and a reconfiguration phase. To explore this alternative, we conducted additional analyses targeting short-lived changes in IBS occurring around the time of the disruption.

- **Methods**

We used the same methodology as in the main analysis, but instead of extracting the three IBS features across the entire 10-minute pre- and post-disruption phases, we focused on a narrower time window.

- - ***Identification of the appropriate window size***

The window size was determined based on behavioral data. Average error score signals were computed separately for dyads in the control and experimental groups. A distinct peak in error scores was observed approximately 25 seconds after the disruption in the experimental group, which was absent in the control group (see **Figure SM-6**). This peak was interpreted as an indicator that the teams were effectively challenged by the disruption. The subsequent decrease in errors was considered to mark the onset of adaptation. Accordingly, the local analysis window was set to span 25 seconds before and after the disruption event.


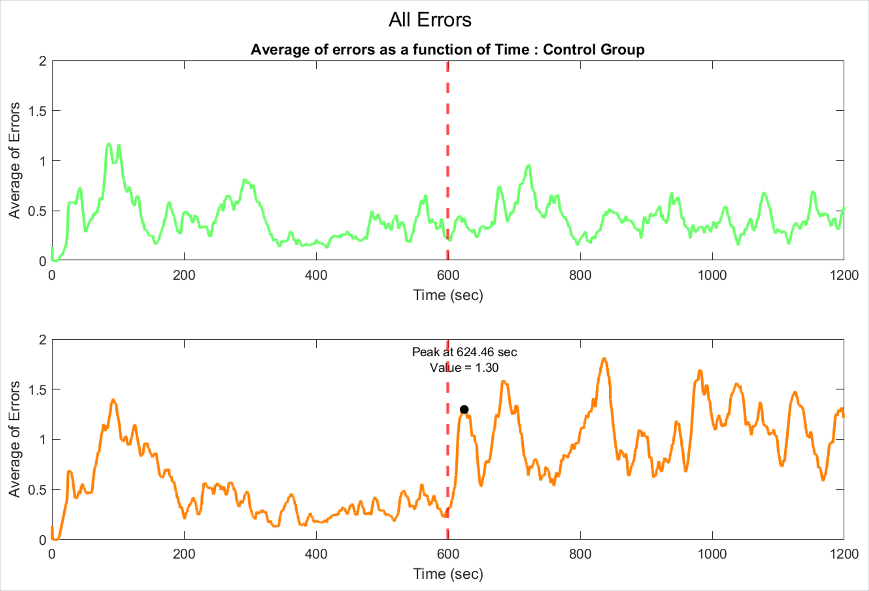


**Figure SM-6:** Errors as a function of time in Control (top) and Experimental (bottom) groups. The peak of errors appears at 24.46 sec after the disruption.

- - ***Statistical analyses***

The same analyses as in the main analyses were used (i.e., testing the change in IBS level, IBS slope and IBS %DET, before and after the disruption in control and experimental groups, using linear mixed effect models).

- **Results**

The results of the three ANOVAs conducted on the linear mixed-effects models are presented in **Table SM-6**. Regarding IBS level, the ANOVA comparing models with and without the interaction term was statistically significant. However, the fixed effect of the Group × Time interaction was not significant in the model summary, suggesting that the interaction may not have a strong or consistent influence across ROIs. For both Slope and %DET, neither the ANOVA nor the fixed effects were significant. These analyses indicate that there were no significant local effects of the disruption on IBS dynamics.

**Table SM-6:** Results of Linear Mixed Models: local effect of disruption on IBS (level, slope and %DET).

| **ANOVA** | | | | | | | | |  | **Fixed effect** | | | |
| --- | --- | --- | --- | --- | --- | --- | --- | --- | --- | --- | --- | --- | --- |
| **Feature** | **Model** | **npar** | **AIC** | **BIC** | **logLik** | **χ²** | **df** | **p** |  | **Coeff** | **[95% IC]** | **p** | **mR²** |
| **Level** | Null | 6 | -1454 | -1428 | 733 |  |  |  |  |  |  |  |  |
|  | Reduced | 7 | -1458 | -1427 | 736 | 5.31 | 1 | 0.021 |  | 0.015 | [-0.012; 0.042] | 0.271 | 0.02 |
|  |  |  |  |  |  |  |  |  |  |  |  |  |  |
| **Slope** | Null | 6 | -7529 | -7503 | 3771 |  |  |  |  |  |  |  |  |
|  | Reduced | 7 | -7530 | -7499 | 3772 | 2.44 | 1 | 0.118 |  | 0.000 | [-0.000; 0.000] | 0.119 | 0.03 |
|  |  |  |  |  |  |  |  |  |  |  |  |  |  |
| **DET** | Null | 6 | -885 | -859 | 449 |  |  |  |  |  |  |  |  |
|  | Reduced | 7 | -886 | -856 | 450 | 3.30 | 1 | 0.069 |  | -0.034 | [-0.070; 0.003] | 0.070 | 0.01 |

Note: npar = number of parameters; AIC = Akaike Information Criterion; BIC = Bayesian Information Criterion; logLik = log-likelihood; df = degrees of freedom; mR² = marginal R²
